# Characteristics and Outcomes of Women With Mild and Moderate Forms of COVID-19 Giving Birth During the COVID-19 Pandemic

**DOI:** 10.7759/cureus.41397

**Published:** 2023-07-05

**Authors:** Sema Baki Yıldırım, Dilek Yeniay

**Affiliations:** 1 Department of Obstetrics and Gynecology, Giresun University, Giresun, TUR; 2 Department of Anesthesiology and Reanimation, Giresun Maternity and Child Health Training and Research Hospital, Giresun, TUR

**Keywords:** pandemic, obstetric, gynecology, pregnancy, covid-19

## Abstract

Introduction: We aim to investigate the clinical course and impact of mild and moderate forms of coronavirus disease 2019 (COVID-19) infection on pregnant women.

Method: A retrospective cohort study was conducted on pregnant women who delivered in a hospital with confirmed COVID-19 infection. Demographic features, clinical characteristics, and perinatal outcomes were retrospectively evaluated.

Results: In total, 157 pregnant women with COVID-19 were hospitalized. In a total of 46 deliveries, three (6.5%) had comorbidities and six (13%) were symptomatic. Myalgia and cough were the leading symptoms. In total, 11 (23.8%) patients received COVID-19 therapy, 41 (90%) had mild disease, and five (10.9%) were transferred to the intensive care unit (ICU). Maternal mortality was observed in two (4.3%) cases. Of the patients, 15 (32.6) had pregnancy complications (preterm delivery) (n = 13, 28.2%), and the cesarean section rate was 91.3%.

Conclusion: The course of COVID-19 was mild in the majority of cases. However, accompanying comorbid conditions may accelerate the return to severe form and cause death.

## Introduction

The coronavirus disease 2019 (COVID-19) is caused by the severe acute respiratory syndrome coronavirus 2 (SARS-CoV-2) [1-3]. Since its emergence, both the response to the virus and the virus have had devastating effects on world health, economies, and societies [1]. Previously, data were limited on the effects of COVID-19 on the fetus and pregnant women.

During pregnancy, changes in the immune, cardiac, pulmonary, and other systems that occur in a pregnant woman’s body can increase the risk of infection and its associated mortality and morbidity [1,3-5]. Understanding the susceptibility to infection during pregnancy is difficult, as in addition to susceptibility, the level of exposure to the pathogen is also important [1,6]. In a prospective cohort analysis of disease occurring between adults of childbearing age and pregnant women at the same time period, the frequency of infection during pregnancy was similar to US-modeled estimates [4]. Present data do not support the notion that pregnancy can increase susceptibility to COVID-19 infection, but conclusions are inconsistent. Studies defining whether pregnancy increases the risk of serious illness are limited; however, subsequent research comparing nonpregnant women of childbearing age with pregnant women suggested that pregnancy is a risk factor for serious illness [4-7]. It has been stated that COVID-19 may cause an increase in obstetric complications such as preterm labor and fetal distress [8].

Moreover, during pregnancy, several risk factors for serious illness have been identified, such as pre-pregnancy comorbid conditions, higher maternal age, and high body mass index (BMI) [1,8]. A meta-analysis and systematic review showed that pregnant and newly pregnant women, in comparison with nonpregnant women of childbearing age, have increased odds of admission to the intensive care unit (ICU) and increased need for invasive ventilation and extracorporeal membrane oxygenation [6,8].

There is controversial data about the course of COVID-19 in pregnant women [1]. The main objective of this research was to evaluate the clinical signs and maternal and perinatal outcomes of pregnant women with mild and moderate forms of COVID-19.

## Materials and methods

This retrospective cohort study was conducted on pregnant women with confirmed COVID-19 infection whose deliveries occurred between March 11, 2020, and October 30, 2021. All patients accepted into the study were in the third trimester, and all of them had mild and moderate forms of COVID-19 and were hospitalized in Giresun University Maternity and Children’s Hospital. Pregnant women in their first or second trimesters and with severe form of COVID-19 were followed up in a different center as per protocol. They were excluded from the study. (According to the decision taken by the health authorities of our region, pregnant women outside the third trimester and severe COVID-19 cases were followed in another hospital with advanced intensive care conditions.) In this study, the data used were collected from inpatient medical records. Pregnancies with negative real-time polymerase chain reaction (RT-PCR) results for SARS-CoV-2 were excluded from the study.

Nasopharyngeal swabs were taken from all pregnant women and then tested for definitive diagnosis of COVID-19 infection using reverse transcriptase real-time PCR. The following were then retrospectively recorded: maternal age, parity, gravidity, pre-pregnancy body mass index (BMI) (calculated as weight in kilograms divided by the square of height in meters), gestational age at diagnosis, initial symptoms, time interval between diagnosis and delivery, route of delivery, medicines linked to COVID-19, respiratory support, admission to the intensive care unit (ICU), maternal mortality, initial laboratory test result, and blood group. Blood samples were taken from the patient upon admission to the hospital. In all pregnant women, fetal ultrasonography (USG) measurements of biparietal diameter, femur length, and abdominal circumference were performed. Patients were either asymptomatic or had varying degrees of clinical manifestations. They were classified as mild, moderate, and severe forms of COVID-19 according to the severity of symptoms and test results. A mild form of COVID-19 is characterized by fever, cough, sore throat, fatigue, and a body temperature below 38.5°C. A moderate form of COVID-19 is characterized by fever (38.5°C and above), a respiratory rate of more than 22 per minute, oxygen saturation of more than 95%, and shortness of breath during physical activity. COVID-19 is considered severe if the respiratory rate is more than 30 per minute, oxygen saturation is 94% and lower, PaO2/FiO2 is 300 mmHg and lower, there is a progression of lung damage (an increase of damaged tissue by 50% or more within 24-48 hours), consciousness decreased, hemodynamics are unstable, arterial blood lactate is more than 2 mmol/L, and the quick Sequential Organ Failure Assessment (qSOFA) score is more than 2 [1].

Statistical analysis

Statistical analyses were performed using the Statistical Package for the Social Sciences (SPSS) version 25 (IBM SPSS Statistics, Armonk, NY, USA). Numerical variables were presented as mean ± standard deviation (SD) and minimum-maximum, and categorical variables were presented as number and percentage as descriptive statistics.

Ethical approval

The study procedure complied with the ethical standards set out in the Helsinki Declaration, and ethics committee approval was obtained from Gumushane Scientific Research and Publication Ethics Committee with its decision dated 09.11.2021 and numbered 2021/7. This study was designed retrospectively, and informed written consent was received from none of the patients.

## Results

This retrospective cohort study was conducted on pregnant women with confirmed COVID-19 infection. In total, 157 pregnant women were hospitalized with a total of 46 deliveries. The clinical characteristics and demographic features of the cases are shown in Table 1.

**Table 1:** Demographic features and clinical characteristics of pregnant women with COVID-19 infection (n = 46) Values are given as number (percentage), mean ± SD (range), or median (IQR/range). Abbreviations: BMI, body mass index; COVID-19, coronavirus disease 2019; ICU, intensive care unit; SD, standard deviation; IQR, interquartile range

Demographic features and clinical characteristics (n = 46)	
Maternal age (years)	32.02 ± 4.6 (20-40)
Gravidity	2.19 (1-6)
Parity	0.9 (0-3)
Pre-pregnancy BMI (kg/m^2^)	24.9 ± 3.51 (18-33)
Initial symptoms (%)	
Asymptomatic	40 (87)
Symptomatic	6 (13)
COVID-19 severity	
Mild	41 (90)
Moderate	5 (10)
Comorbid disease	3 (6.5)
Hypertension	1 (2.1)
Epilepsy	1 (2.1)
Gestational diabetes	1 (2.1)
COVID-19 therapy	
Low-molecular-weight heparin	46 (100)
Systemic corticosteroid	8 (17.3)
Lopinavir-ritonavir	3 (6.5)
Respiratory support	
Nasal oxygen therapy	5 (10.8)
High-flow nasal cannula	0 (0)
Invasive mechanical ventilation	0 (0)
Transfer to ICU	5 (10.9)
Maternal mortality	2 (4.3)

Forty (87%) cases were asymptomatic. Myalgia and cough were the leading symptoms. Low-molecular-weight heparin was administered in 46 (100%) cases. Five (10.9%) cases were transmitted to the ICU. Nasal oxygen support was given to five (10.8%) patients. Maternal mortality was observed in two (4.3%) cases. The first case of maternal death was a 33-year-old primipara at 28 weeks of pregnancy, who has a history of previous myocardial infarction and was admitted to cesarean section with the indication of preterm premature rupture of membranes and twin pregnancy with regular uterine contractions. On admission, she had no symptoms. She was transmitted to the intensive care unit due to postoperative dyspnea on the first day of hospitalization. Afterward, the patient died on the fourth day of her admission to the intensive care unit due to multiple organ failure. The second case of maternal mortality was a 38-year-old primipara at 31 weeks of pregnancy who underwent a cesarean section due to fetal distress. She was sent to the ICU due to postoperative respiratory distress and decrease in oxygen saturation and then died on the 18th postoperative day. These two patients were also taken to cesarean section for feto-maternal reasons, not COVID-19 infection. The first laboratory test results of pregnant women with COVID-19 infection are shown in Table 2.

**Table 2:** Initial laboratory test results of pregnant women with COVID-19 infection (n = 46) Values are given as number (percentage) or mean ± SD (range). Abbreviations: ALT, alanine aminotransferase; AST, aspartate aminotransferase; BUN, blood urea nitrogen; COVID-19, coronavirus disease 2019; CRP, C-reactive protein; ESR, erythrocyte sedimentation rate; Hb, hemoglobin; Hct, hematocrit; SD, standard deviation

Variables	Values
Hb (g/dL)	11.5 ± 1.6 (8.3-15.3)
Hct (%)	35 ± 3.8 (26-43)
Hb < 10 mg/dL	11 (23.9)
Leukocyte (10³/mm³)	8,993 ± 3,524 (4,060-19,800)
Leukocytosis (>11,000/mm³)	9 (19.6)
Neutrophil (10³/mm³)	7,108 ± 3,141 (2,300-17,000)
Neutrophilia (>7,700/mm³)	14 (30.4)
Lymphocyte (10³/mm^3^)	1,375 ± 724 (500-3,280)
Lymphocytopenia (<1,000/mm³)	17 (37)
Neutrophil-to-lymphocyte ratio	6.07 ± 3.5 (1.30-19.5)
Platelet (10³/mm³)	200,762 ± 62,367 (122,000-414,000)
CRP (mg/dL)	26 ± 27.3 (1.2-125)
CRP increase (>10 mg/ dL)	26 (56.5)
ESR (mm/hour)	45.7 ± 20 (14-96)
Procalcitonin (ng/mL)	0.11 ± 0.15 (0-0.80)
Ferritin (ng/mL)	58.1 ± 59 (5.7-246)
BUN (mmol/L)	14.5 ± 6.5 (4-30)
Creatinine (mg/dL)	0.4 ± 0.13 (0.20-0.80)
ALT (IU/L)	42.04 ± 171 (5-1,173)
AST (IU/L)	35.7 ± 78 (9-524)
Elevated liver enzymes (ALT and AST ≥2 times the upper limit)	2 (4.3)
LDH (IU/L)	232.4 ± 68.2 (154-515)
D-dimer (mcg/mL)	1,863 ± 1,494 (74-7,685)
D-dimer increase (>600 ng/L)	34 (73.9)
Potassium (mmol/L)	3.9 ± 0.38 (3-5)
Radiologic imaging	6 (13)
Radiologic imaging findings suspicious for COVID-19	5 (10.8)
Blood type	
A+	21 (45.6)
A-	1 (2.17)
B+	8 (17.3)
AB+	2 (4.34)
0+	14 (30.4)

Neutrophilia and lymphocytopenia were observed in 14 (30.4%) and 17 (37%) cases, respectively. Elevated liver enzymes were observed in two (4.3%) patients. No hypokalemia was present in the cases. Radiologic imaging was performed for six (13%) patients. All patients had radiologic imaging findings suspicious for COVID-19. A Rh+ and 0 Rh+ type blood groups were the most common (45.6% and 30.4%, respectively). The obstetric outcomes of pregnant women with COVID-19 are shown in Table 3.

**Table 3:** Obstetric outcomes of pregnant women with COVID-19 (n = 46) Values are given as number (percentage), mean ± SD, and minimum-maximum. Abbreviations: COVID-19, coronavirus disease 2019; SD, standard deviation

Obstetric outcomes (n = 46)	
Time interval between diagnosis and delivery (days)	4.12 ± 1.76 (1-13)
Preterm delivery	13 (28.2)
COVID-19-indicated preterm delivery	3 (6.5)
Pregnancy complications	15 (32.6)
Gestational hypertension	1 (2.1)
Preterm delivery	13 (28.2)
Preeclampsia	1 (2.1)
Route of delivery (number (%))	
Normal spontaneous vaginal delivery	4 (8.7)
Cesarean delivery	
Previous cesarean delivery	23 (50)
Primary cesarean delivery	19 (41.3)
Cesarean indications (number (%))	
Previous cesarean delivery	23(50)
Maternal health condition	2 (4)
Cephalopelvic disproportion	6 (13)
Fetal distress	4 (8)
Multiple pregnancies	1 (2)
Due to COVID-19 infection	6 (13)
Gestational age at delivery (weeks)	36.8 ± 3.1 (28-41)
Birth weight (g)	2,950 ± 738 (935-3,965)

Preterm delivery was observed in 13 (28.2%) patients, three of which had COVID-19 indications. The rate of cesarean delivery was 91.3%; six of them were due to COVID-19 infection. Previous cesarean delivery was the most common indication for cesarean delivery (n = 23, 50%), and cesarean section was performed in four (8%) cases owing to fetal distress. Spontaneous vaginal delivery accounted for four (8.7%) cases. The mean gestational age at delivery and birth weight were 36.8 ± 3.1 weeks and 2,950 ± 738 g, respectively.

## Discussion

Managing complicated pregnancies due to COVID-19 infection in the first days of the pandemic was challenging for doctors because there is no consensus yet on the subject as regards the optimal treatment method, route of delivery and timing, choice of the optimal imaging method, and indications for hospitalization [8]. However, the National Institutes of Health has developed guidelines for the care and treatment of COVID-19 patients (https://www. covid19treatmentguidelines.nih.gov/) [9].

This study population was predominantly young, and 6.5% had major comorbidities. The relationship between COVID-19 infection and concomitant disease has been known for a long time [10]. There are various identified risk factors for severe disease during pregnancy; these are conditions such as advanced maternal age, increased BMI, pre-pregnancy hypertension, and diabetes [1,5]. Despite the mild course of COVID-19 in most patients, the presence of a concomitant chronic illness or being overweight may facilitate viral contamination. The vital signs of most patients (87%) were normal when admitted to the hospital. The most frequent presenting symptoms were cough and myalgia. In pregnant women, the condition of early symptoms has differed among studies, based on clinical, social, and regional factors [8,11,12]. Therefore, overall suspected cases should be thoroughly evaluated.

Increased proportions of obstetric complications (e.g., fetal distress, preterm birth, gestational diabetes, preeclampsia, and cesarean delivery) have been reported in the literature for pregnant women infected with COVID-19 [1-3]. It has been observed that iatrogenic and spontaneous preterm birth rates increase in pregnancies infected with COVID-19. In addition, fetal perfusion disorder and fetal distress may develop due to decreased oxygen saturation, which may result in increased cesarean delivery ratios [3]. However, it is hard to find definite etiologic agents [8]. Previous publications stated that COVID-19 diagnosis increases the risk of cesarean section, although according to the present systematic review, COVID-19 infection was not associated with an increased risk of cesarean delivery. However, the association between preterm birth continued [1,13-15]. Of all patients, 90% have mild COVID-19, 10.9% were transferred to the ICU, and 4.3% died. Pregnancy complications were observed in 32.6% of the cases, and most of the complication was preterm delivery. Nineteen patients underwent cesarean delivery due to primary cesarean indication, and six were due to COVID-19 infection. Our cesarean rate was high. In the early time of the pandemic, there was a high level of fear and stress, as well as the fear of vertical transmission; the high cesarean rate can be explained by this. The differences in the literature on the risks of preterm birth and cesarean delivery may be associated with different obstetric practices among populations, geographic regions, and adaptation to underlying medical conditions [1,16].

COVID-19 is not an indication for delivery and should alter neither the timing nor the mode of delivery in general [5]. Timing of birth should be individualized for pregnant patients, by calculating the risks and benefits for the fetus and patient. Delivery may be considered in a patient with refractory hypoxemia or worsening or insistent crucial disease at or after 32 weeks of gestation [1,17,18]. In our study, cesarean delivery due to COVID-19 was performed in six cases (13%), three of which were preterm.

Clinical algorithms are generally similar for the care of nonpregnant and pregnant women with COVID-19; however, there are some important differences in the use of pregnancy-specific algorithms, such as peripheral oxygen saturation needs to be continued at or above 95% during pregnancy to ensure adequate oxygenation across the placenta. The recommended treatment (e.g., dexamethasone, monoclonal antibodies, and remdesivir) for the nonpregnant population should also be given to pregnant women [1]. In this study, these drugs were used in only a few cases, and the general approach was conservative.

Although patients and doctors have concerns about the safety of COVID-19 drugs during pregnancy, a significant reduction in mortality and morbidity has been reported when the drug is started in the early phase of the disease [19,20]. In the present study, 6.5% of patients received specific treatment for COVID-19. In the research population, systemic corticosteroids and low-molecular-weight heparin were frequently used. In general, systemic corticosteroids, lopinavir, and ritonavir were used in patients with a poor prognosis [8]. Venous thromboembolism prophylaxis is suggested for pregnant women hospitalized with COVID-19 infection [21]. All pregnant women received low-molecular-weight heparin in the present study. In our study populations, noninvasive oxygen support was applied as a primary approach in patients with respiratory distress due to COVID-19 infection, in parallel with the literature [22].

The main determinants of the severe COVID-19 process in pregnancy are decreased levels of lymphocytes and erythrocytes, electrolyte imbalance, and an increase in alanine aminotransferase (ALT) and C-reactive protein (CRP) levels. More research is needed to better understand the mechanism. Moreover, in some cases of maternal death, hypokalemia was one of the first findings. The main reason for maternal death due to COVID-19 was the presence of a serious comorbidity [22]. In the present study, none of the cases had hypokalemia; this may be because there is no severe form of COVID-19. The relationship between the course of COVID-19 and ABO blood types was researched in diverse studies. A and B type blood groups were found to be related to higher rates of SARS-CoV-2 positivity compared to O type blood group [23]. In the present study, O Rh+ and A Rh+ type blood groups were the most common.

The strengths of this study were the large number of study parameters and the single-center practice. However, the main limitation is its retrospective design and small scale, not including all pregnant women.

Since there is no COVID-19 intensive care unit in our hospital, patients with severe COVID-19 were sent to another hospital. Therefore, we could not follow the intensive care processes of the patients.

## Conclusions

The course of COVID-19 was mild in the majority of cases. Even if the form of COVID-19 was mild or moderate, accompanying comorbid conditions may accelerate the return to severe form and cause death.
